# Acute pontine infarction inducing REM sleep behavior disorder: a novel case report

**DOI:** 10.3389/fnhum.2025.1614797

**Published:** 2025-09-23

**Authors:** Liuqing Wang, Hong Wang

**Affiliations:** ^1^Department of Neurology, Nanjing Gaochun People’s Hospital, Nanjing, China; ^2^Department of Neurology, The First Affiliated Hospital of Wenzhou Medical University, Wenzhou, China

**Keywords:** rapid eye movement (REM) sleep behavior disorder (RBD), loss of physiological muscle atonia during REM sleep, dream-enacting behaviors, acute pontine infarction, dysarthria, left-sided limb numbness and weakness, nocturnal behavioral disturbances, left hemiparesis

## Abstract

Rapid eye movement (REM) sleep behavior disorder (RBD) is parasomnia characterized by the loss of physiological muscle atonia during REM sleep, resulting in dream-enacting behaviors that often manifest as complex, violent motor activity. While RBD may occur idiopathically, it is frequently associated with neurodegenerative disorders, particularly *α*-synucleinopathies such as Parkinson’s disease. However, secondary RBD linked to acute cerebrovascular events remains poorly documented. Here, we present a novel case of acute pontine infarction precipitating RBD, highlighting the brainstem’s critical role in REM sleep regulation and expanding the spectrum of secondary RBD etiologies. This case report underscores the importance of neuroanatomical localization in evaluating acute-onset RBD, particularly in the context of cerebrovascular pathology.

## Introduction

**Figure 1:**
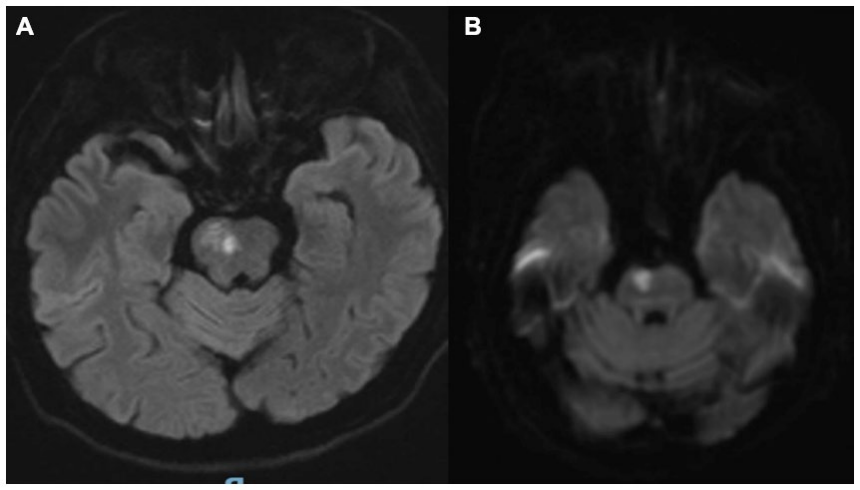
**(A,B)** Brain MRI confirmed an acute ischemic lesion involving the right pontine tegmentum and midbrain.

A 62-year-old woman presented to our institution with a 5-day history of dysarthria, left-sided limb numbness and weakness, and nocturnal behavioral disturbances. The patient initially reported isolated left finger numbness upon awakening, without accompanying dizziness or motor deficits. Over the subsequent 72 h, her condition progressed to include dysarthria, left hemiparesis (Medical Research Council grade 3/5), and intermittent dysphagia, accompanied by hypersomnolence and recurrent nocturnal episodes of disorganized vocalizations, agitated shouting, and complex motor behaviors during sleep. Neuroimaging (MRI) demonstrated an acute infarction involving the right pontine tegmentum and rostral midbrain (Figure 1). Her medical history was significant for longstanding hypertension (≥10 years), poorly controlled (mean BP 165/95 mmHg pre-admission), with no prior history of parasomnias or neurodegenerative symptoms. We conducted a structured interview with the patient’s spouse using the Innsbruck RBD Inventory (Stiasny-Kolster et al., 2010), which revealed that the patient had no history of dream-enactment behaviors prior to the onset and no observed nocturnal vocalizations or complex movements before disease onset.

### Physical examination and diagnostic findings

On admission, the patient was drowsy (Glasgow Coma Scale 12/15) with a blood pressure of 125/70 mmHg. Cranial nerve assessment revealed intact extraocular movements in all planes, preserved facial sensation, symmetrical forehead wrinkling, and full sternocleidomastoid/trapezius strength. Neurological examination demonstrated left hemiparesis (Medical Research Council grade 3 + in upper/lower limbs) with preserved muscle tone and proprioception. The left Babinski sign was equivocal. Cardiopulmonary auscultation revealed irregular rhythm (heart beat rate: 80 bpm) consistent with pre-existing atrial fibrillation, without pulmonary abnormalities.

### Neuroimaging and vascular studies

Brain MRI confirmed an acute ischemic lesion involving the right pontine tegmentum and midbrain (Figures 1A,B). Transcranial Doppler and cervical duplex ultrasonography identified right vertebral artery occlusion, reduced flow velocities in the left vertebral artery (28 cm/s vs. normal >40 cm/s), and the basilar artery (35 cm/s), along with bilateral carotid intima-media thickening (1.2 mm) containing non-stenotic heterogeneous plaques.

### Polysomnographic documentation

Video-polysomnography captured three REM sleep episodes with pathognomonic features: ① Sustained phasic electromyographic hyperactivity in submentalis and tibialis anterior leads. ② Episodic vocalizations and complex motor sequences (arm thrusting, pedaling motions) lasting 110–240 s. ③ Complete absence of REM-atonia index (RAI = 0%, normative >90%). The polysomnographic recordings (Philips Alice 6) were performed using the international 10–20 system with 19 scalp electrodes, including standard placements at F3, F4, C3, C4, O1, and O2 for REM sleep scoring. A referential montage was used with linked mastoid references (A1 + A2), maintaining electrode impedances below 5 kΩ throughout the recordings.

To exclude the possibility of neurogenesis, olfactory function assessed by the Sniffin’ Sticks test was normal (score: 13/16; normosmia cutoff >12). Autonomic evaluation revealed no abnormalities, with negative results for constipation (Rome IV criteria) and orthostatic hypotension. Due to institutional resource constraints and cost considerations, DAT-SPECT imaging was not performed in this case.

The patient was initiated on clonazepam 0.5 mg nightly beginning on the second day of admission, which resulted in partial symptom control. Follow-up polysomnography at 2 months demonstrated improvement in REM atonia. However, melatonin therapy was not administered in this case due to institutional medication availability constraints, though we suppose that melatonin may be more effective and safer.

## Discussion

Rapid-eye-movement sleep behavior disorder (RBD), first characterized by Schenck et al. in 1986, exhibits a population prevalence of approximately 0.38%, rising to 0.5% among adults aged ≥60 years (Schenck et al., 1987). The disorder demonstrates a striking male predominance (male-to-female ratio ~9:1), though emerging evidence suggests under-recognition in females due to phenotypic variability (Gros and Videnovic, 2020). While RBD onset spans adulthood, its incidence peaks sharply after the sixth decade, with fewer than 5% of cases occurring in individuals under 40 years (Dauvilliers et al., 2018).

RBD demonstrates a well-established association with *α*-synucleinopathies, including Parkinson’s disease (PD), multiple system atrophy (MSA), and dementia with Lewy bodies (DLBs) (Malkani, 2023). Emerging evidence suggests RBD may also manifest secondary to various neurological conditions, encompassing cerebrovascular disorders, intracranial neoplasms, demyelinating diseases, and neurovascular abnormalities (Postuma et al., 2019; Shinno et al., 2010; Foschi et al., 2019; Matsumoto and Tsunematsu, 2021). Pediatric RBD cases frequently present with comorbid neurological pathologies, particularly epilepsy spectrum disorders and brainstem tumors (Shukla et al., 2020; McCarter et al., 2015). The disorder exhibits considerable variability in symptom frequency, ranging from isolated episodes (≤1/month) to multiple nightly events. Notably, disease progression typically correlates with increased event frequency and behavioral complexity, potentially reflecting underlying neurodegenerative processes. The differential diagnosis of RBD should include three principal mimics: Nocturnal epileptic seizures manifesting as stereotyped movements distinct from RBD’s complex behaviors (Montagna, 1992); severe central sleep apnea (CSA) with arousal-related movements unaccompanied by dream enactment (Thalhofer and Dorow, 1997); and periodic limb movement disorder (PLMD) featuring rhythmic rather than purposeful motor activity (John et al., 2025).

Emerging evidence demonstrates a complex, reciprocal interaction between sleep disorders and cerebrovascular disease. Sleep disorders are not only one of the risk factors for cerebrovascular diseases, but may also be a consequence of cerebrovascular diseases (Gottlieb et al., 2021; Kojic et al., 2022). After suffering stroke, the patient exhibited a triad of post-stroke neurological manifestations: (1) focal sensorimotor deficits, (2) excessive daytime sleepiness (Epworth Sleepiness Scale score: 16/24), and (3) complex nocturnal behaviors including oneiric delirium, vocal outbursts, and violent motor activity correlating with dream content (Kojic et al., 2022). In our case, prior to the emergence of REM sleep behavior symptoms, the patient exhibited pre-existing speech difficulties and left-sided limb weakness. Concurrently, family members reported observing abnormal nocturnal behaviors. This temporal profile supports the structural disruption of the dorsolateral pontine tegmentum as the underlying mechanism. Polysomnography (PSG) monitoring showed increased electromyographic activity during the REM sleep stage, which is consistent with the diagnosis of RBD (Gottlieb et al., 2021). Cerebrovascular diseases can present as symptoms or complications. Sleep disorders after a stroke include excessive sleepiness, difficulty falling asleep, reduced total sleep time, circadian rhythm disruption, central/obstructive apnea, and abnormal sleep-stage behaviors. In this case, the patient developed excessive sleepiness and REM sleep behavior abnormalities after the onset of the disease.

RBD can be an isolated phenomenon, known as idiopathic or sporadic RBD, and can also be associated with nervous system diseases and systemic diseases, known as secondary RBD. The generation and regulation of REM sleep are related to the brainstem (Gros and Videnovic, 2020). The loss of muscle tone is associated with the locus coeruleus and subcoeruleus nucleus (LC/SubLC) (Boeve, 2013). Nerve impulses sent from these nuclei inhibit spinal motor neurons through the medulla oblongata and spinal reticular formation, resulting in the relaxation of skeletal muscles other than respiratory muscles. Disruption of this pathway, whether through neurodegenerative processes or structural lesions, results in the pathological preservation of muscle tone during REM sleep (REM sleep without atonia) and the characteristic dream-enacting behaviors of RBD (Iranzo et al., 2021). Previous literature data show that the midbrain region ventrolateral periaqueductal gray (vlPAG) is known to be important for gating REM sleep (Weber et al., 2018), we suppose that Ischemic damage to the SLD-vlPAG pathway may contribute to the RBD in this case. The acute symptom onset followed by gradual improvement over 3 months suggests functional compensation, potentially mediated by contralateral pedunculopontine nucleus hyperactivity—a hypothesis that could be confirmed through future fMRI studies. However, idiopathic RBD (iRBD) and secondary RBD (current vascular case) show distinct clinical and prognostic profiles. iRBD is characterized by an underlying *α*-synuclein pathology, insidious symptom onset over years, and isolated REM without atonia on polysomnography. It carries a high risk of neurodegenerative conversion (80% within 10 years) (Postuma et al., 2019) and typically responds well to melatonin or clonazepam. In contrast, the current secondary case resulted from a pontine stroke, with acute symptom onset within 72 h. Consequently, this form requires urgent acute intervention to prevent progression.

Current research suggests that the functional impairment of the pontine-midbrain neural structures related to REM sleep generation is the pathological mechanism of RBD. In this case, the patient’s infarct was located in the pontine tegmentum and midbrain, which may have damaged the REM-related nuclei and led to RBD, further supporting the association between pontine - midbrain neural structures and the occurrence of RBD. In recent years, cases of brainstem lesions combined with RBD have been reported in multiple sclerosis, cerebrovascular diseases, tumors, and brainstem inflammation. Additionally, there are reports of RBD associated with limbic encephalitis (Kucukali et al., 2024).

In summary, RBD is closely related to neurodegenerative diseases. However, it was previously thought that RBD rarely occurred in other nervous system diseases. Research on the pathogenesis of REM sleep and RBD has found that RBD is not uncommon in brainstem lesion diseases, which is related to the damage to brainstem nuclei that regulate REM sleep structures. Clinicians should pay attention to this pathological phenomenon of RBD, proactively identify its etiologies, and administer timely treatment.

## Data Availability

The datasets presented in this article are not readily available because of ethical and privacy restrictions. Requests to access the datasets should be directed to the corresponding authors.
